# Evolução da Captação Miocárdica de ^18^F-FDG em Paciente com Diagnóstico de Cardiotoxicidade

**DOI:** 10.36660/abc.20230276

**Published:** 2024-02-16

**Authors:** Diego Rafael Freitas Berenguer, Monica de Moraes Chaves Becker, Roberto de Oliveira Buril, Paula Araruna Bertão, Brivaldo Markman, Simone Cristina Soares Brandão

**Affiliations:** 1 Universidade Federal de Pernambuco Programa de pós-graduação em Saúde Translacional Recife PE Brasil Programa de pós-graduação em Saúde Translacional–- Universidade Federal de Pernambuco, Recife, PE – Brasil; 2 Universidade Federal de Pernambuco Programa de pós-graduação em Cirurgia Recife PE Brasil Programa de pós-graduação em Cirurgia – Universidade Federal de Pernambuco, Recife, PE – Brasil; 3 Universidade Federal de Pernambuco Hospital das Clínicas de Pernambuco Recife PE Brasil Hospital das Clínicas de Pernambuco – Universidade Federal de Pernambuco, Recife, PE – Brasil

**Keywords:** Cardiotoxicidade, Linfoma, Tomografia por Emissão de Pósitrons combinada à, Tomografia Computadorizada, Insuficiência Cardíaca, Doxorrubicina

## Abstract

O objetivo deste relato é mostrar a evolução da cardiotoxicidade (CTX) por quimioterápicos em paciente com linfoma por exames de imagens, destacando a importância da captação miocárdica de flúor-18 fluordeoxiglicose (^18^F-FDG) pela tomografia por emissão de pósitrons, acoplada à tomografia computadorizada (PET/CT).

Feminino, 43 anos, com linfoma uterino, submetida a histerectomia, três esquemas de quimioterapia (QT), sucessivamente, e radioterapia. Apresentou episódios de insuficiência cardíaca aguda dois anos após QT. Ecocardiograma mostrou redução da fração de ejeção do ventrículo esquerdo (FEVE). Análise retrospectiva do ^18^F-FDG PET/CT observou elevação da captação miocárdica em todos os exames durante o seguimento oncológico.

Apesar da remissão oncológica, a paciente desenvolveu IC com FEVE reduzida. Durante a QT, ocorreu aumento difuso e significativo da captação miocárdica de ^18^F-FDG, que precedeu a queda do desempenho cardíaco, e pareceu refletir alterações metabólicas nos cardiomiócitos relacionadas à CTX. A análise da captação miocárdica de ^18^F-FDG modificaria o desfecho cardiológico da paciente? Esse questionamento é relevante, visto que outros pacientes podem se beneficiar desse método como marcador precoce de CTX.

Os exames de imagem são imprescindíveis no acompanhamento de pacientes com risco de CTX. O ecocardiograma permanece como principal auxílio diagnóstico, porém o ^18^F-FDG PET/CT pode estar surgindo como uma poderosa ferramenta para um diagnóstico mais precoce dessa condição clínica.

## Descrição

Paciente feminino, 43 anos, com linfoma difuso de grandes células B uterino, estágio IIIB, submetida à histerectomia seguida de quimioterapia (QT) e radioterapia (RT). Como antecedente, possuía o diagnóstico de doença renal crônica em terapia de substituição renal (TSR), devido à ureterohidronefrose bilateral pela doença primária uterina. Ecocardiograma pré-QT com hipertrofia ventricular esquerda (HVE) concêntrica e fração de ejeção do ventrículo esquerdo (FEVE) preservada (66%).

A paciente foi submetida a oito ciclos do esquema terapêutico R–CHOP (Rituximabe, Ciclofosfamida, Doxorrubicina, Vincristina). Por refratariedade ao tratamento primário, realizou terapia de resgate com esquema R-ICE (Mesna, Ifosfamida, Etoposídeo, Carboplatina) quatro ciclos, porém apresentou intolerância a Carboplatina. O tratamento foi alternado para GEMOX (Oxaliplatina, Gencitabina) e finalizado após quatro ciclos. Foi então submetida a 28 sessões de RT e, desde então, não apresenta sinais de recidiva oncológica.

Dois anos após o início da QT, foi internada com quadro de insuficiência cardíaca (IC). Novo ecocardiograma evidenciou FEVE de 35%. Cintilografia de perfusão miocárdica mostrou redução da FEVE (37%) pelo *gated SPECT* e hipoperfusão transitória nas paredes apical e inferosseptal do ventrículo esquerdo (VE). A cineangiocoronariografia não evidenciou doença arterial coronariana epicárdica.

Desde então, ocorreram quatro internamentos por descompensação da IC, o último há um ano. Ressonância magnética cardíaca (RMC) evidenciou FEVE de 39% e realce tardio, não isquêmico, em parede inferolateral do VE.

No momento, a paciente encontra-se estável, em uso de Carvedilol 25mg/dia, Losartan 100mg/dia e Furosemida 40mg/dia. A hipótese diagnóstica de cardiotoxicidade (CTX) foi considerada a mais provável como etiologia da IC com FEVE reduzida (ICFER).

### Evolução dos exames de imagem cardiológica

**Figura 1:**
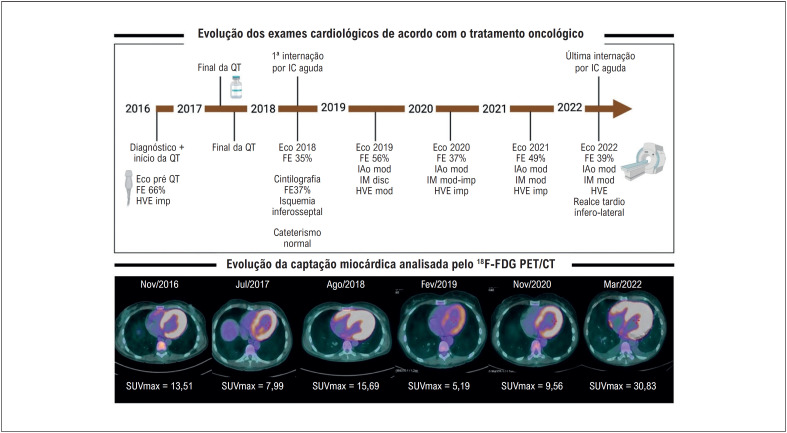
Evolução dos exames cardiológicos e da captação miocárdica de fluordeoxiglicose-flúor-18 (^18^F-FDG) na tomografia por emissão de pósitrons, acoplada à tomografia computadorizada (PET/CT) de acordo com o tratamento oncológico recebido. QT: quimioterapia; Eco: ecocardiograma; FE: fração de ejeção; HVE: hipertrofia ventricular esquerda; imp: importante; RT: radioterapia; IC: insuficiência cardíaca; IAo: insuficiência aórtica; IM: insuficiência mitral; disc: discreta; mod: moderada; int: internação; RM: ressonância magnética; Nov: novembro; Jul: Julho; Ago: Agosto; Fev: Fevereiro; Mar: Março; SUV: standard uptake value; max: máximo. Imagem confeccionada com Biorender.com.

Em virtude da evolução do caso, os exames de imagem cardíaca foram alocados cronologicamente, para melhor entendimento (Figura 1). Observa-se uma queda da FEVE no ecocardiograma realizado no momento do diagnóstico da IC. Essa FEVE teve uma recuperação no ano de 2019, com novos decréscimos nos ecocardiogramas seguintes, assim como na RMC.

Em avaliação retrospectiva da captação miocárdica de fluordeoxiglicose-flúor-18 (^18^F-FDG) pela tomografia por emissão de pósitrons, acoplada à tomografia computadorizada (PET/CT), nota-se aumento da captação desde o primeiro exame, realizado após o início da QT. O *standard uptake value* (SUV) máximo permaneceu elevado nos exames subsequentes, atingindo seu maior valor no último exame.

## Discussão

O diagnóstico de CTX persiste como importante desafio na prática clínica. No caso acima, CTX é a hipótese mais provável apesar das poucas informações prévias à QT.

Mesmo com perfil de risco moderado para desenvolvimento de CTX da paciente (dose elevada de antracíclico, HVE por provável hipertensão arterial e TSR),^1^ o primeiro ecocardiograma foi realizado apenas dois anos após o início da QT. Medidas terapêuticas para tratamento da ICFER só foram instituídas quando houve descompensação clínica da IC. Nesse sentido, destaca-se a necessidade de uma interação multidisciplinar para maior prevenção de complicações no cuidado ao paciente oncológico.^2^

Exames de PET/CT com ^18^F-FDG são realizados rotineiramente nos pacientes com linfoma.^3^ No caso relatado, após análise retrospectiva dos exames, foi identificado um aumento da captação miocárdica de ^18^F-FDG. E qual a relevância clínica do aumento da captação cardíaca de ^18^F-FDG durante e pós-QT? Apesar da resposta definitiva não estar clara na literatura, estudos sugerem que este aumento pode ser um indicador precoce de CTX.^4-9^

Borde et al.^4^ observaram que, em pacientes com linfoma tratados com doxorrubicina (doses >250mg/m²), o aumento na captação miocárdica de ^18^F-FDG poderia ser um marcador precoce de CTX. Outro estudo com 69 pacientes com linfoma de Hodgkin demonstrou aumento progressivo na captação cardíaca de ^18^F-FDG, durante tratamento, que persistiu seis meses após término da QT.^5^

Estudo em pacientes com linfoma Hodgkin e esquema quimioterápico primário contendo doxorrubicina, demonstrou que o SUV de ^18^F-FDG do VE aumentou gradualmente ao longo da QT. Ademais, quando os pacientes foram categorizados em alto ou baixo SUV, a FEVE foi significativamente mais baixa naqueles com SUV de VE elevado.^6^

Uma avaliação de 121 pacientes com câncer de mama que realizaram ^18^F-FDG PET/CT oncológico e ecocardiograma pré-QT demonstrou que, ao final do tratamento, o grupo que desenvolveu CTX tendeu a apresentar aumento da captação de ^18^F-FDG no VE associado a padrão difuso de hipercaptação. Esse estudo também mostrou associação significativa da captação de ^18^F-FDG no ventrículo direito com o desenvolvimento de CTX.^7^

Dourado et al.^8^ observaram elevação significativa da captação miocárdica de ^18^F-FDG nos pacientes submetidos à QT por linfoma. Em mais da metade dos pacientes, o SUV máximo no miocárdio ventricular esquerdo aumentou mais de 30% pós-QT, comparando-se com o exame pré-QT.

É válido salientar que o padrão miocárdico de captação de ^18^F-FDG pode apresentar certa variabilidade e os limites de normalidade desse padrão ainda não são estabelecidos. Fatores como sexo, idade, alimentação rica em carboidratos nos dias anteriores ao exame, obesidade, diabetes e algumas medicações podem influenciar o consumo miocárdico de ^18^F-FDG.^9^ Apesar disso, existem padrões de captação miocárdica considerados fisiológicos.^10^

No caso descrito, com aumento progressivo do SUV miocárdico, pode-se considerar a paciente como seu próprio controle. Esse aumento se manifesta de forma diferente do estabelecido até o momento como captação fisiológica.^10^

Sendo assim, a análise da captação miocárdica de ^18^F-FDG alteraria o desfecho cardiológico dessa paciente? Esse questionamento é relevante, dado que outros pacientes podem se beneficiar do uso da PET como marcador precoce de CTX. As informações de aumento da captação, no início da terapia, poderiam levar a uma estratégia de cardioproteção mais efetiva, com possível melhora da sobrevida e redução da morbimortalidade.

## Conclusão

O ecocardiograma mantém-se como o principal exame de imagem no diagnóstico de CTX, porém a ^18^F-FDG PET/CT pode ser uma poderosa ferramenta para seu diagnóstico mais precoce.
