# Supplementary material for: Evolução da Captação Miocárdica de 18F-FDG em Paciente com Diagnóstico de Cardiotoxicidade
Source: Arq Bras Cardiol. 2024 Feb 16;121(2):e20230276. [Article in Portuguese] doi: 10.36660/abc.20230276 (PMC11081128; doi:10.36660/abc.20230276)
Supplement: Translation

# Translation: Progression of Myocardial ^18^F-FDG Uptake in a Patient with Cardiotoxicity

**Authors:** Diego Rafael Freitas Berenguer, Monica de Moraes Chaves Becker, Roberto de Oliveira Buril, Paula Araruna Bertão, Brivaldo Markman, Simone Cristina Soares Brandão

**Affiliations:** 1 Recife PE Brasil Programa de pós-graduação em Saúde Translacional –- Universidade Federal de Pernambuco, Recife, PE – Brazil; 2 Recife PE Brasil Programa de pós-graduação em Cirurgia – Universidade Federal de Pernambuco, Recife, PE – Brazil; 3 Recife PE Brasil Hospital das Clínicas de Pernambuco – Universidade Federal de Pernambuco, Recife, PE – Brazil

**Keywords:** Cardiotoxicity, Lymphoma, Positron Emission Tomography Computed Tomography, Heart Failure, Doxorubicin

## Abstract

The objective of this case report was to present the progression of chemotherapy-induced cardiotoxicity in a patient with lymphoma, highlighting the importance of myocardial fluor-18-fluorodeoxyglucose (18F-FDG) uptake by positron emission tomography coupled with computed tomography (PET/CT).

43-year-old female patient with uterine lymphoma, who underwent hysterectomy followed by three chemotherapy regimens and radiotherapy. The patient had episodes of acute heart failure two years after chemotherapy. Echocardiogram revealed a reduction in left ventricular ejection fraction (LVEF). A retrospective analysis of ^18^F-FDG PET/CT showed an increase in myocardial uptake in all tests performed during oncologic treatment.

Despite disease remission, the patient developed heart failure with reduced LVEF. During chemotherapy, there was a diffuse, significant increase in myocardial ^18^F-FDG uptake, which preceded the decrease in myocardial performance and seemed to reflect metabolic changes in cardiomyocytes, related to cardiotoxicity. Would an analysis of myocardial ^18^F-FDG uptake yield a different cardiac outcome in this patient? This question is relevant, considering that other patients may benefit from the use of PET as an early marker of cardiotoxicity.

Imaging tests are essential in the follow-up of patients at risk of cardiotoxicity. Although echocardiography remains the main imaging test in the diagnosis of cardiotoxicity, 18F-FDG PET/CT may be a powerful tool for the early diagnosis of this condition.

## Description

A 43-year-old female patient with uterine diffuse large B-cell lymphoma, stage 3B, who underwent hysterectomy followed by chemotherapy and radiotherapy. The patient had a history of chronic renal disease and renal replacement therapy (RRT) due to bilateral hydroureteronephrosis caused by the primary uterine disease. Pre-chemotherapy echocardiogram showed concentric left ventricular hypertrophy (LVH) and preserved left ventricular ejection fraction (pLVEF) (66%).

The patient underwent eight cycles of R-CHOP regimen (rituximab, cyclophosphamide, vincristine, doxorubicin, and prednisone). As the patient was refractory to the primary treatment, the patient received salvage therapy with four cycles of the R-ICE protocol (Mesna, ifosfamide, carboplatin, and etoposide phosphate), but was intolerant to carboplatin. The treatment was changed to GEMOX (oxaplatin, gemcitabin) and finished after four cycles. Then the patient underwent 28 radiotherapy sessions and had no signs of oncologic recurrence since then.

Two years after the onset of chemotherapy, the patient was admitted with heart failure (HF). The echocardiogram showed a LVEF of 35%. Myocardial perfusion scintigraphy showed a reduction in the LVEF (37%) by gated SPECT imaging and a transient hypoperfusion of apical and inferoseptal walls of the left ventricle. Cineangiography showed no evidence of epicardial coronary disease.

Since then, the patient had four admissions for HF decompensation, the last one occurring one year ago. Cardiac magnetic resonance (CMR) showed LVEF of 39% and non-ischemic, and late enhancement of left ventricular inferolateral wall.

The patient is currently stable, taking 25mg/d carvedilol, 100mg/d losartan and 40mg/d furosemide. Cardiotoxicity was the most likely cause of HF with reduced LVEF.

## Time course of cardiac Imaging results

Cardiac imaging results were placed in a chronological order to better understand the course of this case (Figure 1). A reduction in LVEF is observed in the echocardiogram performed at the diagnosis of HF. There was a recovery of the LVEF in 2019, with new reductions in the following echocardiograms, as well as in the CMR.

**Figure 1:** Time course of cardiac imaging results and myocardial F-18-fluorodeoxyglucose (18F-FDG) uptake on positron emission tomography/computed tomography (PET/CT) by oncologic treatment. LVH: left ventricular hypertrophy; CTX: chemotherapy; RTX: radiotherapy; EF: ejection fraction; AoI: aortic insufficiency; MI: mild insufficiency; Nov.: November; Jul.: July; Aug.: August, Feb.: February; Mar: March; SUV: standard uptake value; max.: maximum. Image created using Biorender.com.

A retrospective analysis of myocardial uptake of F-18-fluorodeoxyglucose (^18^F-FDG) by positron emission tomography/computed tomography (PET/CT) showed an increase in the uptake since the first test during chemotherapy. The maximum standard uptake value (SUV) remained high in the following tests and reached the highest value in the last test.

## Discussion

The diagnosis of cardiotoxicity is still a challenge in the clinical practice. In this reported case, cardiotoxicity is the most likely hypothesis although few baseline (before chemotherapy) data were available.

Although the patient had a moderate risk of cardiotoxicity (high dose of anthracycline, LVH due to probable arterial hypertension, and RRT),^1^ the first echocardiogram was performed only two years after chemotherapy was initiated. Therapeutic measures for HF with reduced LVEF were only implemented in the presence of clinical decompensation of HF. In this regard, it is important to highlight the need for a multidisciplinary approach to improve prevention in the management of oncologic patient.^2^

^18^F-FDG PET/CT imaging is routinely performed in patients with lymphoma.^3^ In the present case, a retrospective analysis of the tests revealed increased myocardial uptake of ^18^F-FDG. And what is the clinical relevance of such increase in cardiac uptake of ^18^F-FDG during and after chemotherapy? Although there is no definite answer in the literature, studies have suggested that this increase may be an early indicator of cardiotoxicity.^4-9^

Borde et al.^4^ reported that, in lymphoma patients treated with doxorubicin (doses >250mg/m²), enhanced myocardial ^18^F-FDG uptake may be an early marker of cardiotoxicity. Another study with 69 patients with Hodgkin disease showed a progressive increase in myocardial ^18^F-FDG uptake during treatment that persisted six months after the end of chemotherapy.^5^

A study with Hodgkin disease and primary chemotherapy with doxorubicin showed that left ventricular ^18^F-FDG SUV gradually increased during chemotherapy. In addition, when patients were categorized as low and high SUV, LVEF was significantly lower in those with high left ventricular SUV.^6^

An analysis of 121 breast cancer patients who underwent oncologic FDG PET/CT and echocardiography before chemotherapy showed that, at the end of treatment, those patients who developed cardiotoxicity tended to show enhanced, more diffuse left ventricular ^18^F-FDG uptake. This study also showed a significant association between right ventricular ^18^F-FDG uptake and cardiotoxicity.^7^

Dourado et al.^8^ observed a significant increase in myocardial ^18^F-FDG uptake in patients undergoing chemotherapy for lymphoma. In more than half of patients, a percentage increase greater than 30% of left ventricular maximal SUV occurred after chemotherapy as compared with before chemotherapy.

It is worth pointing out that myocardial pattern of ^18^F-FDG uptake may vary, and the normal threshold of this pattern has not been established yet. Factors like sex, age, high carbohydrate intake on the days prior to the exam, obesity, diabetes, and some medications may influence myocardial ^18^F-FDG uptake.^9^ Despite that, there are patterns of myocardial uptake that may be considered physiological.^10^

In this reported case, with the progressive increase in myocardial SUV, the patient can be considered as her own control. Such increase differs from what has been established so far as physiological uptake.^10^

That being said, would an analysis of myocardial ^18^F-FDG uptake yield a different cardiac outcome in this patient? This question is relevant, considering that other patients may benefit from the use of PET as an early marker of cardiotoxicity. Detection of increased uptake in the beginning of therapy could lead to a more effective cardioprotective strategy, with potential improvement in survival and reduction in morbidity and mortality.

## Conclusion

Although echocardiography remains the main imaging test in the diagnosis of cardiotoxicity, 18F-FDG PET/CT may be a powerful tool for an early diagnosis of this condition.
